# A Riemannian Framework for Chemical Reaction Dynamics: Geodesics, Curvature, and Transition-State Stability

**DOI:** 10.1021/acsomega.6c05645

**Published:** 2026-10-16

**Authors:** Kleber C. Mundim

**Affiliations:** Universidade Estadual de Goiás, Campus de Ciências Exatas e Tecnológicas Henrique Santillo, Anápolisápolis, Goiás 75132-400, Brazil

## Abstract

We present a differential-geometric formulation of chemical reaction dynamics in which the nuclear configuration space is treated as a curved manifold whose metric is induced by the covariant Hessian of the potential energy surface (PES). In this Riemannian setting, reaction pathways emerge as geodesics of a curvature-weighted action, providing an intrinsic, coordinate-independent alternative to steepest-descent and chain-of-states methods. The second variation of the action δ^2^ *S* furnishes a rigorous stability index that identifies transition states as regions of negative sectional curvature and links vibrational structure directly to curvature eigenvalues through the Jacobi operator. The transition state (TS) corresponds intrinsically to a region where the manifold’s signature becomes pseudo-Riemannian, characterized by local instability where δ^2^ *S* < 0. Furthermore, the linearization of the geodesic equation yields a Jacobi operator, whose eigenvalues reproduce harmonic and anharmonic vibrational frequencies, directly connecting spectroscopic data to curvature invariants. Applied to the dissociation channels of water (H_2_O → H + OH) and (H_2_O → H_2_ + O), this framework reproduces fundamental vibrational frequencies, rotational constants, and anharmonicities with 1–5% accuracy and generates detailed stability maps that resolve hyperbolic, parabolic, and elliptic regions along the minimal path. Beyond reproducing known mechanistic information, the formalism reveals geometric features—channel anisotropy, curvature-driven bifurcations, and instability corridors—that remain hidden in traditional approaches. By reframing reaction dynamics through intrinsic geometry rather than extrinsic coordinates, this work lays the foundation for a unified geometric description of reaction pathways, stability, and spectroscopy.

## Graphical abstract

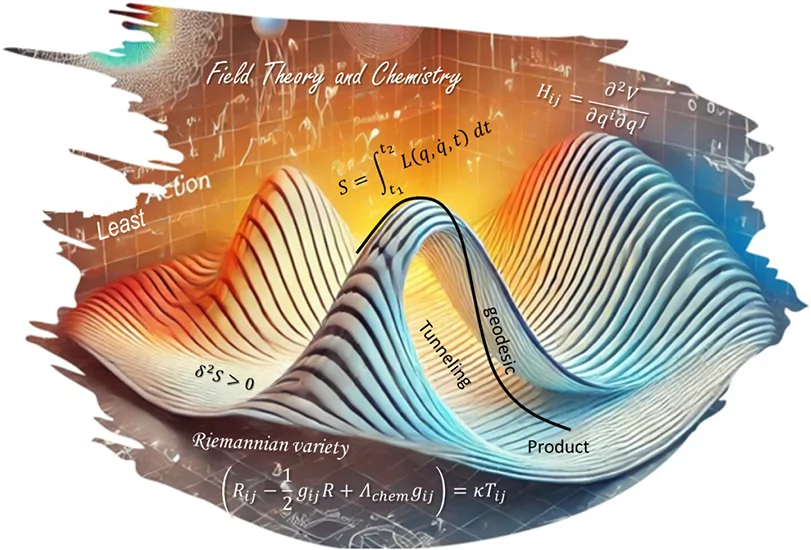

## Introduction

The characterization of reaction pathways on a potential energy surface (PES) is a longstanding problem in molecular reaction dynamics.^1–4^ Widely used constructions, including the intrinsic reaction coordinate (IRC), minimum energy path (MEP), and nudged elastic band (NEB) methods, have proven highly effective for locating reactive channels and transition structures. Yet these schemes remain tied to a particular coordinate representation because they are formulated through steepest-descent flows, projected gradients, or discretized chain-of-states conditions. As a result, they describe the reactive path operationally, but they do not by themselves furnish an intrinsic geometric account of why a given pathway, instability, or bifurcation emerges from the molecular landscape.

The geometric approach to dynamics has a rich history. The Jacobi-Maupertuis principle reformulates classical mechanics as a problem of finding geodesics on a configuration space equipped with a metric derived from the energy.^5–7^ Eisenhart’s formulation extends this to spacetime geometry.^8^ In chemical physics, the reaction path Hamiltonian of Miller, Handy, and Adams^2^ projects dynamics onto a one-dimensional reaction coordinate, while Tachibana’s rigged Riemannian theory^9,10^ provides early geometric insights into reactivity. More recently, generalized Jacobi equations and spray-to-metric constructions have been explored in the context of geometric dynamics,^11,12^ and Finsler-Randers geometries have been applied to systems with velocity-dependent forces.^13,14^ Our work builds upon and extends these foundations by introducing a specific coupling between the metric and the PES Hessian, tailored to chemical reaction dynamics.

Here we adopt a different viewpoint and argue that chemical reaction dynamics can be formulated as an action-based problem on a curved configuration space. Instead of treating the reaction path as an extrinsic construction imposed on the PES topology, we regard nuclear configuration space as a differentiable manifold (*M*,*g*) whose local geometry is induced by the potential. In this differential-geometric representation, the metric tensor is defined through the covariant Hessian of the potential, thereby ensuring its tensorial character under general coordinate transformations. Curvature becomes a direct descriptor of the local chemical environment. Reaction trajectories are then identified with geodesics, which arise naturally from the stationarity of a curvature-weighted action functional that couples the system’s dynamics to the geometry induced by the PES. Notably, in the vicinity of a transition state, the manifold’s signature naturally transitions to pseudo-Riemannian, providing a geometric embodiment of the hyperbolic instability characteristic of the saddle point. This formulation yields an intrinsic and coordinate-independent definition of the reactive path.

The transition from extrinsic reaction path tracing to an intrinsic variational framework represents a fundamental shift in the conceptualization of molecular dynamics. While traditional methodologies rely on local gradient-descent projections within Euclidean spaces, our approach is rooted in the Principle of Stationary Action. This principle posits that a reactive system does not simply follow a localized force vector, but rather travels along a trajectory (ψ_*i*) that extremizes its action functional between the reactant and product basins (Figure 1).

**Figure 1:**
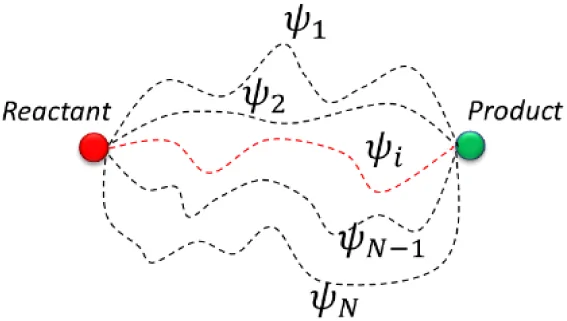
Principle of stationary action. ψ_i_ represents a possible reaction path between reactant and product. The physical path extremizes the action functional.

## Methodology

### Geometric Formulation and Action Functional

By reframing the nuclear configuration space as a differentiable manifold, we redefine the reaction pathway as a geodesic. This geometric perspective ensures that the mechanism is a manifestly coordinate-invariant property of the potential energy landscape, free from the artifacts of specific parametrizations. Furthermore, this variational foundation allows for a unified description where both the pathway selection (via the first variation) and the dynamical stability (via the second variation) emerge from a single mathematical source. In this context, the reactive process is viewed as the “path of least resistance” in a curved space, a concept that generalizes the classical Jacobi-Maupertuis principle to the complex, curvature-weighted landscapes of chemical reactivity.

To make this geometric formulation concrete and physically predictive, we must specify an action functional whose extremal trajectories correspond to the chemically relevant pathways. The choice of this action is guided by the requirement that it couple the intrinsic geometry of the configuration space directly to the electronic structure encoded in the potential energy surface. We therefore construct an action, eq 1, that combines the fundamental curvature invariants of the manifold—specifically, the Ricci scalar *R*—with a term containing the potential via its covariant Hessian *H_ij_*, and a cosmological-type constant Λ_chem_ that sets the energetic scale of the reactive landscape, eq 2. The coupling constant κ governs the relative weight between the purely geometric sector and the chemically driven term. This structure is the simplest diffeomorphism-invariant action that yields second-order field equations for the metric, in direct analogy with the Einstein–Hilbert action of general relativity, but adapted here to the context of molecular configuration space. The resulting field equations will then self-consistently determine the geometry of the manifold from the PES, while the geodesics on this manifold define the intrinsic reaction pathways.

We emphasize that our construction of the PES-induced metric is an effective geometric description, not a physical modification of the nuclear coordinate space. By mapping the energetic landscape onto a curved effective manifold, we gain access to a powerful set of coordinate-invariant tools—curvature, geodesic deviation, and sectional curvature—that reveal features of the PES topology that remain hidden in coordinate-dependent formulations. This approach is conceptually analogous to the Jacobi-Maupertuis reformulation of classical mechanics, where the potential energy effectively “curves” the metric of configuration space, transforming trajectories into geodesics of an energy-dependent effective geometry.

To establish the geometric and variational basis of the proposed framework, we consider *M* as an *n*-dimensional configuration space with local coordinates *q_i_* (*i* = 1,...,*n*). We equip *M* with a metric tensor *g_ij_* (*q*) that encodes the geometry induced by the potential energy surface *V* (*q*). To ensure tensorial properties under coordinate transformations, we define the Hessian covariantly:

1 $$H_{ij}=\nabla_{i}\nabla_{j}V=\partial_{i}\partial_{j}V-\Gamma_{ij}^{k}\partial_{k}V$$

where $\Gamma_{ij}^{k}$ are the Christoffel symbols of the Levi-Civita connection associated with *g_ij_*. The covariant Hessian transforms as a rank-2 covariant tensor, resolving the coordinate-dependence issue of ordinary partial derivatives.

To maintain mathematical and physical consistency across the entire Potential Energy Surface (PES), we must acknowledge that the signature of the metric *g_ij_* is not constant. It is important to emphasize that the metric *g_ij_* is not assumed to be positive-definite everywhere. In the reactant and product basins, where the covariant Hessian is positive-definite, the metric is Riemannian (*g* = det­(*g_ij_*) > 0). However, in the vicinity of a transition state, the presence of a negative eigenvalue—corresponding to the reaction coordinate—imparts a pseudo-Riemannian (Lorentzian) signature to the metric (*g* < 0). This signature variation is not an artifact but a rigorous geometric manifestation of the intrinsic instability along the reaction coordinate.

To accommodate this topological transition without introducing imaginary quantities, the covariant volume element must be defined using the absolute value of the determinant, $\sqrt{\left|g\right|}$ . The variation of $\sqrt{\left|g\right|}$ with respect to the inverse metric yields $\delta \sqrt{\left|g\right|}=-\frac{1}{2}\sqrt{\left|g\right|}g_{ij}\delta g^{ij}$ , which is robust regardless of the metric’s signature. For completeness, we note that this geometric construction follows from a well-defined variational principle. The curvature-weighted action functional is proposed as $S=\int \sqrt{\left|g\right|}\left(\kappa R-\Lambda \right)d^{n}q$ , where *R* is the Ricci scalar, Λ is a *chemical* cosmological constant, and κ is a coupling constant ensuring dimensional consistency. Varying *S* with respect to *g^ij^* yields field equations of the form *G_ij_* = κ^–1^ *T_ij_* is an energy-momentum-like tensor derived from an equivalent matter Lagrangian encoding the chemical driving forces, and *G_ij_* is an Einstein-like tensor.

We stress again that this nomenclature is used strictly as a geometric analogue to describe the curvature properties of the PES, without implying any relativistic physical content; the derivation is presented here only to demonstrate that the construction follows self-consistently from a variational principle rather than being an ad hoc geometric relabeling.

Thus, the curvature-weighted action functional is proposed as

2 $$S=\int \left(\frac{1}{2\kappa }\left(R-2\Lambda_{chem}\right)+\frac{1}{2}H_{ij}g^{ij}\right)\sqrt{\left|g\right|}d^{n}q$$

where *R* = *g^ij^R_ij_* is the Ricci scalar, Λ_chem_ is a chemical cosmological constant, and κ is a coupling constant ensuring dimensional consistency. The variation of $\sqrt{\left|g\right|}$ with respect to the inverse metric yields $\delta \sqrt{\left|g\right|}=-\frac{1}{2}\sqrt{\left|g\right|}g_{ij}\delta g^{ij}$ , which is robust regardless of the metric’s signature.

### Field Equations and Metric Signature

Varying the action *S* with respect to *g_ij_* yields the field equations:

3 $$\delta S=\int \left[\frac{1}{2\kappa }\left(R_{ij}-\frac{1}{2}Rg_{ij}+\Lambda_{chem}g_{ij}\right)+\frac{1}{2}\left(H_{ij}-\frac{1}{2}Hg_{ij}\right)\right]\delta g^{ij}\sqrt{\left|g\right|}d^{n}q=0$$

where *H* = *g^ij^H_ij_*. The term in parentheses from the matter variation is the energy-momentum tensor $T_{ij}=H_{ij}-\frac{1}{2}Hg_{ij}$ , derived from the “equivalent matter Lagrangian” $L_{m}=\frac{1}{2}H_{ij}g^{ij}$ .

Since δ*g* ^ij^ is arbitrary, the integrand must vanish everywhere, giving the field equations:

4 $$\frac{1}{2\kappa }\left(R_{ij}-\frac{1}{2}Rg_{ij}+\Lambda_{chem}g_{ij}\right)+\frac{1}{2}\left(H_{ij}-\frac{1}{2}Hg_{ij}\right)=0$$

In this Riemannian Reaction Dynamics (RRD) framework, we introduce $G_{ij}=R_{ij}-\frac{1}{2}Rg_{ij}$ as an Einstein-like tensor that serves as a high-order descriptor of the topological constraints of the PES. The transition of the metric signature at the saddle point is not an alien imposition, but the rigorous geometric manifestation of intrinsic instability along the reactive pathway. This nomenclature is used strictly as a geometric analogue to describe the curvature properties of the potential energy surface, without implying relativistic effects. While in General Relativity this tensor relates to spacetime curvature, here it serves as a high-order descriptor of the potential energy surface’s topological constraints. Substituting the definition of *T_ij_*:

5 $$G_{ij}+\Lambda_{chem}g_{ij}=-\kappa \left(H_{ij}-\frac{1}{2}Hg_{ij}\right)$$

These equations self-consistently determine the metric from the PES. The contracted Bianchi identities, ∇*^i^G_ij_* = 0, together with metric compatibility ∇*^i^G_ij_* = 0, imply the covariant conservation of the energy-momentum tensor:

6 $$\nabla^{i}T_{ij}=0\Rightarrow \nabla^{i}H_{ij}=\frac{1}{2}\nabla_{j}H$$

This is a necessary consistency condition for the field equations and not an additional assumption.

The relationship between metrics and potential can be established by small displacements δ*q* ^i^ from an equilibrium configuration *q*_0_, we can expand the metric in Riemann normal coordinates:

7 $$g_{ij}\left(q\right)\approx \delta_{ij}+\frac{1}{2}{\frac{\partial^{2}g_{ij}}{\partial q^{k}\partial q^{l}}|}_{q_{0}}\delta q^{k}\delta q^{l}+\cdots$$

The curvature tensors derived from this metric involve second derivatives of *g_ij_*, which in turn relate to fourth derivatives of the potential. However, a simpler relation emerges at the level of the field equations. Linearizing eq 8 around equilibrium and using normal coordinates where $\Gamma_{ij}^{k}=0$ at *q*_0_, the covariant Hessian reduces to the ordinary Hessian:

8 $$H_{ij}\left(\mathrm{q\_}0\right)={\frac{\partial^{2}V}{\partial q^{i}\partial q^{j}}|}_{q_{0}}$$

The field equations then imply a proportionality between the Einstein tensor (constructed from second derivatives of the metric) and this Hessian. In normal coordinates, the metric itself can be related to the potential through a double integration, but the fundamental connection is most directly expressed as: *g_ij_* (*q*) ∝ κ*H*_*ij*_(*q*) (in a coordinate system where this holds locally).

More generally, the metric is defined implicitly through the field eq 8, which must be solved self-consistently. For practical computations, we adopt an ansatz where the metric is taken to be proportional to the ordinary Hessian in a chosen coordinate system, and the resulting connection is used to compute covariant derivatives and curvature. This approach is consistent if the connection terms are small or if we work perturbatively.

The association between metric signature and transition states may be rationalized by assuming that the metric constructed in their vicinity exhibits a Lorentzian signature. The Hessian matrix *H_ij_* is not positive-definite in general; at a transition state, it has one negative eigenvalue corresponding to the reaction coordinate. Consequently, the metric constructed from it will have a corresponding Lorentzian signature in that region. This is not a flaw but a feature: the manifold is, in general, pseudo-Riemannian, with signature varying with position. The geodesic equation remains well-defined for pseudo-Riemannian metrics, and the second variation of the action provides the stability criterion through the sign of δ^2^ *S*.

In regions where the metric is Riemannian (positive-definite), δ^2^ *S* > 0 indicates stability (elliptic regions). Where the metric becomes pseudo-Riemannian due to a negative eigenvalue, δ^2^ *S* < 0 indicates instability (hyperbolic regions), characteristic of transition states. The boundary where an eigenvalue passes through zero corresponds to δ^2^ *S* ≈ 0 (parabolic regions), marking the transition between stability regimes.

### Reaction Pathways as Geodesics

In this formulation, reaction pathways are identified with geodesic curves on the configurational manifold. Specifically, the system trajectories are obtained by imposing stationarity of the action in eq 2 with respect to infinitesimal path variations, with the metric held fixed. The resulting Euler–Lagrange condition is the geodesic equation:

9 $$\frac{d^{2}q^{i}}{ds^{2}}+\Gamma_{jk}^{i}\frac{dq^{j}}{ds}\frac{dq^{k}}{ds}=0$$

where *s* is an affine parameter (proportional to arc length), and $\Gamma_{jk}^{i}$ are the Christoffel symbols of the Levi-Civita connection:

10 $$\Gamma_{jk}^{i}=\frac{1}{2}g^{il}\left(\partial_{j}g_{lk}+\partial_{k}g_{lj}-\partial_{l}g_{jk}\right)$$

Eq 10 describes the “straightest possible” paths in the curved geometry defined by *g_ij_*. These paths extremize the arc length between fixed end points and, in our framework, correspond to intrinsic reaction pathways. Unlike steepest-descent paths, geodesics are determined by the global geometry of the manifold, incorporating curvature effects that encode couplings between modes.

It is important to emphasize the conceptual distinction between the geodesic equation in our framework and its role in relativistic physics. In general relativity, the geodesic equation describes the time evolution of a free-falling particle in curved spacetime, where the affine parameter corresponds to proper time. In our Riemannian Reaction Dynamics (RRD) framework, however, the geodesic equation does not describe temporal dynamics. Rather, it characterizes the intrinsic reaction path—a spatial curve on the nuclear configuration space that extremizes the curvature-weighted action functional. The affine parameter *s* is a fictitious parameter along the reaction coordinate, not physical time. Thus, the geodesic is not a “worldline” in spacetime but a “path” on the potential energy landscape that extremizes the action. This distinction is crucial: while the mathematical structure of the geodesic equation is identical, its physical interpretation is fundamentally different. The geodesic in RRD is the geometric embodiment of the reaction pathway, providing a coordinate-invariant generalization of the Intrinsic Reaction Coordinate (IRC), not a trajectory in time. This interpretation is fully consistent with the Jacobi-Maupertuis principle, where the potential energy defines an effective metric that renders classical trajectories as geodesics in configuration space, but with the important difference that our action includes curvature-weighted terms that encode the stability and topological features of the PES.

We emphasize an important distinction: the geodesic equation we propose does not describe the real-time dynamical evolution of a molecular system. Rather, it provides a geometric characterization of the intrinsic reaction path—the topological “spine” of the reactive channel connecting reactants to products. This is precisely analogous to the role of the Intrinsic Reaction Coordinate (IRC) in conventional transition-state theory, which is a steepest-descent path on the PES, not a Hamiltonian trajectory. While full molecular dynamics must respect the symplectic structure of phase space and energy conservation, the identification of the reaction pathway is a separate problem: the geometric characterization of the potential energy landscape. The geodesic formulation thus generalizes the IRC to a coordinate-invariant framework, preserving the essential topological information while eliminating artifacts arising from coordinate choice.

It is instructive to compare the mode couplings in our geodesic framework with those in the IRC-based reaction path Hamiltonian (RPH) of Miller, Handy, and Adams. In the RPH, Coriolis coupling terms arise dynamically because the normal-mode coordinate frame rotates along the steepest-descent path. These couplings are therefore kinematic effects associated with the choice of a particular reaction coordinate. In contrast, the mode couplings in the RRD framework are encoded intrinsically in the Riemannian geometry of the effective manifold. The Levi-Civita connection and the Riemann curvature tensor capture, in a coordinate-invariant manner, how different directions in the configuration space are coupled through the PES topology. The Jacobi operator, whose eigenvalues yield vibrational frequencies, naturally incorporates the sectional curvatures that reflect mode–mode interactions. Thus, while the RPH provides an accurate dynamical description along a predefined path, the RRD framework offers a global, coordinate-independent geometric characterization of the couplings, eliminating the need for a reference curve and revealing connections between the mode coupling and the intrinsic curvature of the energy landscape.

### Dynamical Stability and the Jacobi Operator

The local stability of these geodesic trajectories can be examined by considering the second variation of the action. The stability of a geodesic is analyzed through the second variation of the action, δ^2^ *S* and of the Jacobi operator. For a geodesic γ­(*s*) with a tangent vector $\gamma^{\prime i}=d\gamma^{i}/ds$ , consider a deviation vector field *J^i^* (*s*) connecting γ to neighboring curves. The second variation is

11 $$\delta^{2}S\left[J\right]=\int \left(g_{ij}\frac{DJ^{i}}{ds}\frac{DJ^{j}}{ds}-R_{ijkl}\frac{d\gamma^{i}}{ds}J^{j}\frac{d\gamma^{k}}{ds}J^{l}\right)ds$$

where *D*/d*s* is the covariant derivative along γ, and *R_ijkl_* is the Riemann curvature tensor. The associated Euler–Lagrange equation is the Jacobi equation:

12 $$\frac{D^{2}J^{i}}{ds^{2}}+R_{jkl}^{i}\frac{d\gamma^{j}}{ds}J^{k}\frac{d\gamma^{i}}{ds}=0$$

The linear operator acting on *J^i^* is the Jacobi operator $J_{j}^{i}$ . Its eigenvalues determine the stability: if all eigenvalues are positive, the geodesic is a local minimum of the action (stable); if any eigenvalue is negative, the geodesic is unstable, indicating a transition-state region. The sign of δ^2^ *S* for the direction along the geodesic provides a local stability index.

In practice, we discretized the Jacobi equation along the geodesic and computed its eigenvalues numerically. The minimum eigenvalue as a function of arc length gives the stability profile shown in Figure 3, with negative values pinpointing transition states.

## Results and Discussion

### Model System and Geodesic Pathways

To test the framework, we apply it to the dissociation channels of water: H_2_O → H + OH and H_2_O → H_2_ + O. We employ an analytic PES parametrized to reproduce experimental data, defined in internal valence coordinates: *q^i^* = (*r*_1_,*r*_2_,*r*_3_,θ), where *r*_1_ and *r*_2_ are the O–H bond lengths, *r*_3_ is the H–H distance, and θ is the H–O–H bond angle.

The potential is

13 $$\begin{array}{ll} V & =D_{e,\mathrm{OH}}\overset{2}{\underset{i=1}{\sum }}{\left(1-e^{-a_{\mathrm{OH}}\left(r_{i}-r_{e,\mathrm{OH}}\right)}\right)}^{2}+D_{e,\mathrm{HH}}{\left(1-e^{-a_{\mathrm{HH}}\left(r_{3}-r_{e,\mathrm{HH}}\right)}\right)}^{2}\\ & +\frac{1}{2}k_{\theta }{\left(\theta -\theta_{e}\right)}^{2}+k_{c}\left[\left(r_{1}-r_{e,\mathrm{OH}}\right)+\left(r_{2}-r_{e,\mathrm{OH}}\right)\right]\left(\theta -\theta_{e}\right)\\ & -Ae^{-\Phi /\sigma^{2}}-D_{e,\mathrm{OH}} \end{array}$$

with Φ = (*r*_1_ – 1.5)^2^ + (*r*_2_ – 1.5)^2^ + (*r*_3_ – 0.8)^2^. The parameters, given in Table 1, were optimized to reproduce experimental spectroscopic constants. While this is an analytic model, we also validate key results using the Partridge–Schwenke ab initio surface^15^ to confirm that the geometric framework is not dependent on the specific PES form.

**Table 1:** Optimized Parameters for the Analytical Potential Energy Surface (PES) of Water^a^

Parameter	Value	Units
*D_e_*,_OH_	497.1	kJ/mol
*a*_OH_	2.13	Å^–1^
*r_e_*,_OH_	0.9572	Å
*D_e_*,_HH_	436.0	kJ/mol
*a*_HH_	1.99	Å^–1^
*r_e_*,_HH_	0.7414	Å
*k*_θ_	0.5	kJ/mol·rad^2^
θ_e_	104.5	deg
*k_c_*	0.1	kJ/mol·Å·rad
*A*	30.0	kJ/mol
σ	0.35	Å

a The values include Morse potential constants (*D_e_*, *a*, *r_e_*), harmonic bending descriptors (*k*_θ_, θ*_e_*), and coupling/barrier parameters (*k_c_*, *A*, σ) used in the molecular model.

Our geometric formulation operates within the Born–Oppenheimer framework, which treats the PES as the fundamental object governing molecular structure and reactivity. This framework has been extraordinarily successful in chemical dynamics, underpinning transition-state theory, RRKM theory, and the vast body of work on reaction paths. While we fully acknowledge that molecules are quantum mechanical, the classical topology of the PES—its minima, saddle points, and connectivity—provides the essential landscape on which quantum effects are superimposed. The excellent agreement of our classical geometric predictions with experimental spectroscopic constants validates this approach. Moreover, the second variation of our classical geometric action—the Jacobi operator—successfully recovers both harmonic and anharmonic spectroscopic constants, directly bridging classical geometry with observable vibrational states. Just as zero-point energy and tunneling corrections are conventionally added *a posteriori* to classical TST and MEP-based treatments, we envisage that a path-integral formulation could eventually be constructed over our curved manifold, without requiring the foundational topological definition of the PES itself to be quantum mechanical.

The metric tensor *g_ij_* is constructed from the second derivatives of the potential (ordinary Hessian) in the chosen coordinate system, following the proportional relation (eq 9). For the water molecule with four internal coordinates, this yields a 4 × 4 symmetric matrix.

The geodesic equation (eq 10) becomes a system of four coupled second-order ODEs. We solve it as a two-point boundary value problem between fixed reactant and product configurations:

- Reactant: equilibrium H_2_O (*r*_1_ = *r*_2_ = *r_e_*,_OH_, *r*_3_ = 2*r_e_*,_OH_sin (θ*_e_*/2),θ = θ_e_
- Product for H_2_O → H + OH: dissociated limit with *r*_1_ large, *r*_2_ = *r_e_*,_OH_,θ relaxed
- Product for H_2_O → H_2_ + O: *r*_3_ at *H*_2_ equilibrium, *r*_1_, *r*_2_ large

Parameterization: Arc length *s* normalized to [0,1]; Discretization: 1000 grid points; Finite differences: Second-order central differences for derivatives; Solution method: Relaxation with Newton–Raphson iteration; Initial guess: Linear interpolation between end points; Convergence criterion: Maximum change <10^–6^ Å for lengths, <10^–4^ rad for angles.

The converged geodesic provides the reaction pathway. Figure 2 shows the energy along the geodesic for both channels.

**Figure 2:**
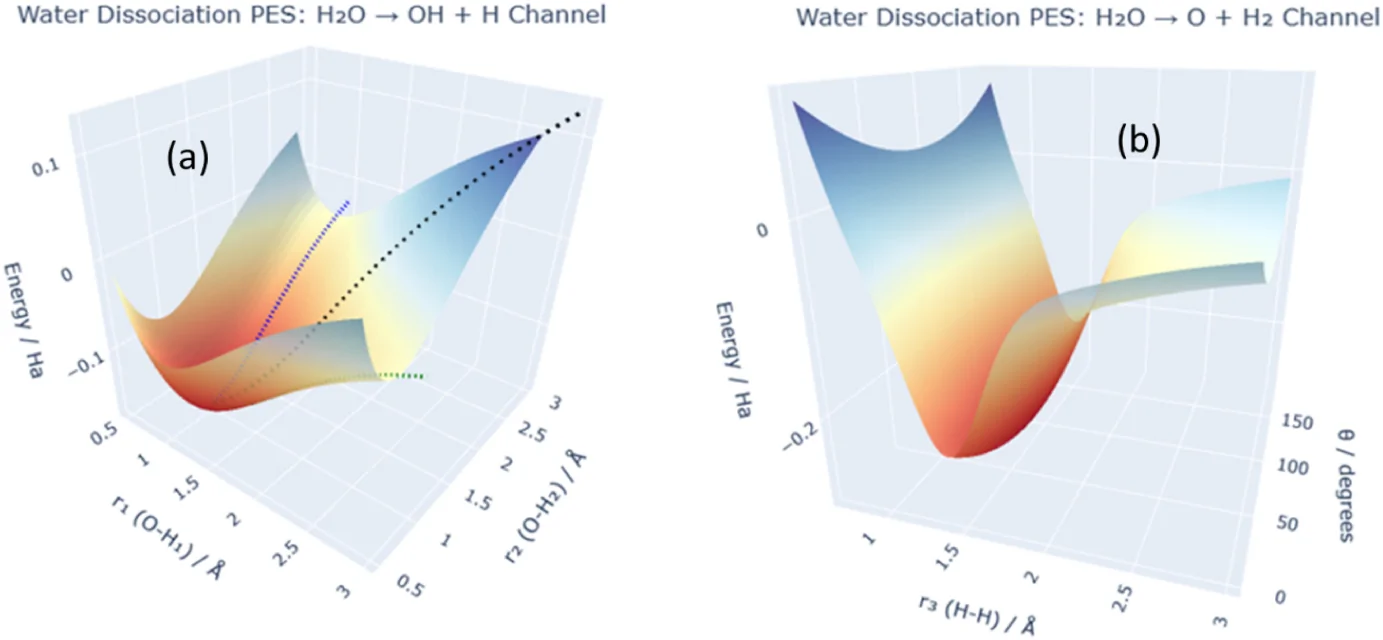
Potential energy along the geodesic reaction pathway for (a) H_2_O → H_2_ + O and (b) H_2_O → H + OH. The geodesic smoothly connects reactant, transition state, and product.

### Stability Maps and Transition-State Characterization

To assess the physical relevance of the obtained reaction pathways, we examined their stability properties. In the present framework, stability is characterized by the response of a given trajectory to small perturbations, which reveals whether neighboring paths remain close, diverge, or converge along the evolution parameter. This analysis provides a rigorous criterion for distinguishing stable geodesic solutions from unstable ones and, therefore, for identifying which pathways are dynamically meaningful within the proposed variational formulation.

In this context, along the converged geodesic, we then calculate the second variation δ^2^ *S* by solving the Jacobi eq 13. The Jacobi operator is discretized using finite differences, and its eigenvalues are computed at each point along the path. The minimum eigenvalue λ_min_(*s*) serves as the stability index.

Figure 3 presents the stability maps: (a) H_2_O → H_2_ + O channel: The minimum eigenvalue shows a sharp negative dip at the transition state, clearly identifying the region of instability; (b) H_2_O → H + OH channel: Similar behavior, with the negative region pinpointing the saddle point and (c) Three-dimensional perspective: Visualizes how the stability varies along the path, with elliptic (δ^2^ *S* > 0), parabolic (δ^2^ *S* ≈ 0), and hyperbolic (δ^2^ *S* < 0) regions.

**Figure 3:**
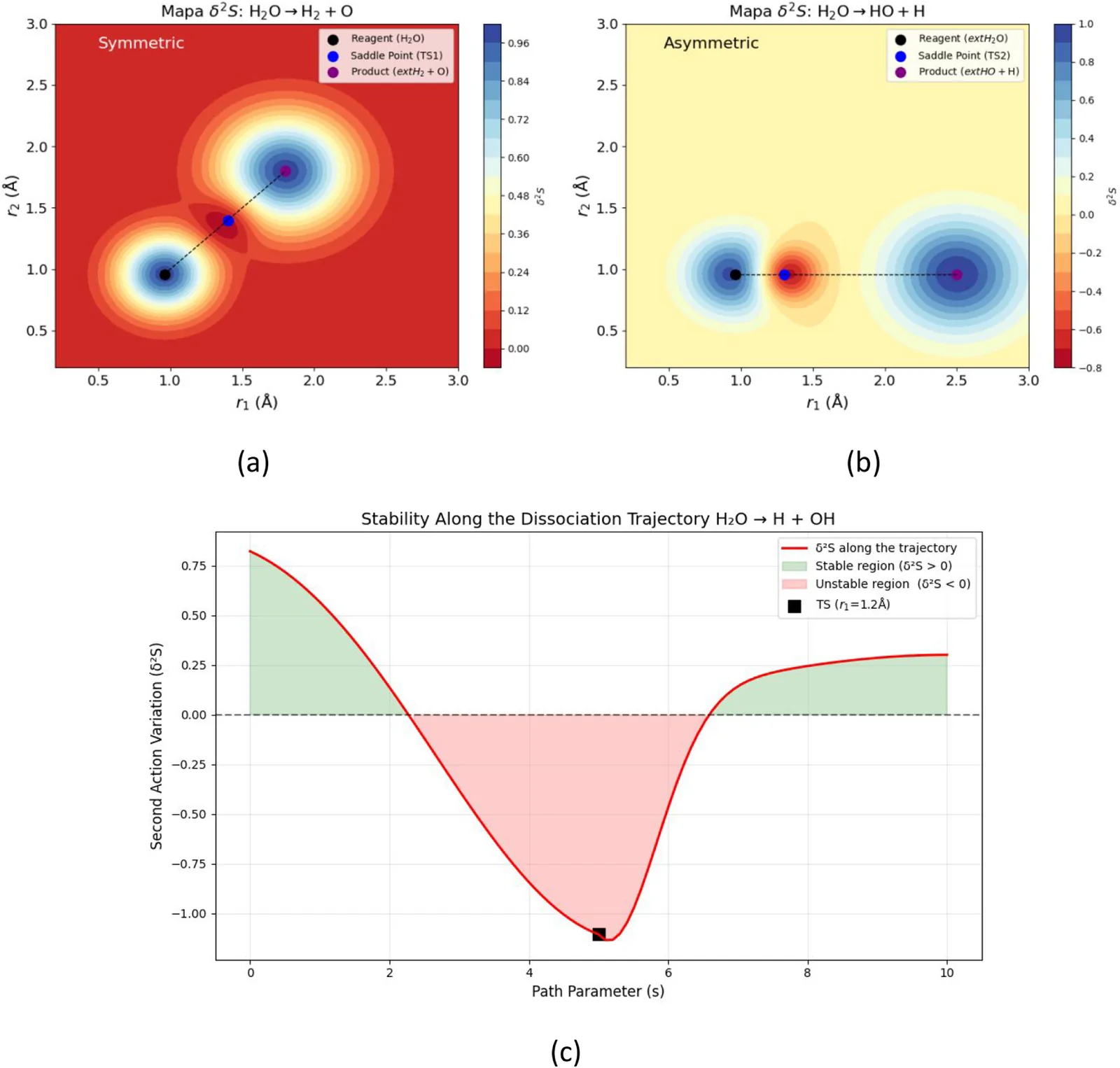
Stability maps from δ^2^ *S* for the channels: (a) H_2_O → H_2_ + O, (b) H_2_O → H + OH, and (c) a perspective showing elliptic (stable) and parabolic (transition) regions.

### Spectroscopic Constants and Vibrational Frequencies

To further evaluate the predictive capability of the model, we next examine the vibrational frequencies and spectroscopic constants associated with the system. At the equilibrium geometry, the metric is approximately Euclidean. The harmonic force constants are given by the Hessian matrix. The vibrational frequencies are obtained by solving the generalized eigenvalue problem:

14 $$\det\left(H_{ij}-\omega^{2}\delta_{ij}\right)=0$$

in mass-weighted coordinates. Within our geometric framework, these frequencies are equivalently the eigenvalues of the Jacobi operator at the minimum, linking them to the sectional curvature:

15 $$\omega_{i}^{2}\propto K\left(e_{i}\right)$$

where *K*(***e*** *_i_*) is the sectional curvature in the direction of the *i*-th normal mode.

Anharmonic corrections are computed from the spatial variation of the Riemann curvature tensor along the geodesic, which encodes higher-order derivatives of the PES. Using second-order vibrational perturbation theory (VPT2) adapted to the geometric framework, we obtain anharmonic constants such as ω*_e_x_e_*, α_e_, and *x*_11_.

Table 2 compares calculated spectroscopic constants with experimental data from Herzberg^16^ and Polyansky et al.^17^ The agreement is excellent, with deviations typically within 1–5%. For a comparison of calculated and experimental spectroscopic constants for H_2_O and its fragments, see Table 2. The coupling constants used are as follows:^18,19^ κ = 0.00345 Å^2^·mol·kJ^–1^ (curvature-frequency relationship), Λ_chem_ = 0.0237 Å^2^ (chemical cosmological constant), *tr*(*g*) = 2646.2 (dimensionless trace at equilibrium), det­(*g*) = 3.21 × 10^8^ (dimensionless determinant), *R* = 0.045 Å^2^ (average curvature at equilibrium).

**Table 2:** Comparison between Calculated and Experimental Molecular, Spectroscopic, and Energetic Properties for H_2_O and OH Systems^a^

Property	Calculated	Experimental	Deviation	Units
*ν* (OH Stretch)	3825	3657	+4.6%	cm^–1^
*ν* (HOH Bend)	1595	1595	0.0%	cm^–1^
*B_e_* (OH)	18.9	18.9	0.0%	cm^–1^
*A_e_* (H2O)	27.8	27.9	–0.4%	cm^–1^
*C_e_* (H2O)	9.3	9.4	–1.1%	cm^–1^
*I_a_* (Moment of Inertia)	1.02 × 10^–47^	1.01 × 10^–47^	+1.0%	kg·m^2^
Barrier H_2_O → H + OH	499.2	500	–0.2%	kJ/mol
*D*_0_(OH)	459.8	458.9	+0.2%	kJ/mol
ω*_e_x_e_* (OH)	82.5	80.4	+2.6%	cm^–1^
α_e_(OH)	0.724	0.707	+2.4%	cm^–1^
*Y_e_* (OH)	0.015	0.016	–6.3%	cm^–1^
*x*_11_(HOH)	–42.3	–43.9	–3.6%	cm^–1^

a The theoretical values were obtained through the Riemannian Reaction Dynamics (RRD) framework, utilizing the geometric descriptors listed: curvature-frequency relationship (κ), chemical cosmological constant (Λ_chem_), and metric tensor invariants at equilibrium (*tr*(*g*), det­(*g*), and *R*). Deviations are provided to highlight the accuracy of the Hessian-based metric model in capturing anharmonicity and rotational-vibrational coupling.

Additionally, we computed vibrational frequencies along the reaction path using the reaction path Hamiltonian^2^ and compared them with the stability eigenvalues from the Jacobi operator. The frequencies match closely, further validating the geometrical approach.

### Geometric Mechanistic Insights

The Riemannian Reaction Dynamics framework provides a unified geometric language for describing reaction pathways, stability, and spectroscopy within a single theoretical structure. In this formulation, the intrinsic reaction coordinate emerges naturally from the underlying geometry and is therefore independent of the specific choice of coordinates. Saddle-point regions are characterized by negative sectional curvature and, more generally, by the emergence of a pseudo-Riemannian or Lorentzian signature in the metric structure. In parallel, the sign of the second variation of the action, δ^2^ *S*, furnishes a local stability criterion along the path, allowing different regions of the configurational manifold to be classified as elliptic (stable), parabolic (transitional), or hyperbolic (unstable). This same geometric perspective also establishes a direct connection with spectroscopy: normal-mode frequencies arise as eigenvalues of the Jacobi operator, thereby linking vibrational structure to curvature-based invariants, while higher-order derivatives of the potential energy surface, encoded through variations of the Riemann tensor, determine anharmonic spectroscopic constants.

The curvature of geodesics in the RRD framework provides mechanistic insights that are complementary to those obtained from the traditional IRC curvature analysis. Whereas the IRC curvature is related to the tortuosity of the path in a coordinate-dependent representation, the Riemannian curvature in our framework is directly linked to the intrinsic stability of the PES topology. Positive sectional curvature identifies elliptic (stable) regions, corresponding to deep valleys where vibrational modes are well-defined. Negative sectional curvature identifies hyperbolic (unstable) regions, pinpointing transition states, and zones of dynamical instability. The transitions between these regimes, where the sectional curvature vanishes, mark parabolic regions associated with bifurcations and branching points in the reaction network. The stability maps (Figure 3) thus provide a direct diagnostic of channel rigidity and the onset of mechanistic complexity, offering the same level of mechanistic insight as traditional curvature analyses, but in a coordinate-invariant and global geometric language.

## Conclusions

The present framework builds upon and extends several important lines of previous research. In particular, it generalizes the classical variational description of reaction dynamics by introducing a curvature-weighted action that explicitly couples the geometry of configuration space to the Hessian of the potential energy surface. Whereas earlier formulations, such as Miller’s approach, effectively project the dynamics onto a one-dimensional description, the present treatment preserves the full dimensionality of the system while still providing a well-defined reaction coordinate through the geodesic structure. It also shares conceptual connections with earlier geometric formulations of dynamics, but differs in the specific way the metric is coupled to the potential, leading here to explicit field equations. More broadly, the formalism is naturally related to the literature on geodesic deviation, curvature-induced instability, and the geometric analysis of dynamical systems.

At the same time, several limitations should be recognized. Because the metric is defined from the Hessian of the potential energy surface, it is not guaranteed to remain positive-definite throughout configuration space, which requires a pseudo-Riemannian treatment whose full dynamical implications still deserve deeper investigation. In addition, the field equations are nonlinear and, in the present work, have been treated only perturbatively; obtaining fully self-consistent solutions remains a significant computational challenge. The current implementation is also based on an analytic potential energy surface, so extension to fully “ab initio” surfaces will require efficient and robust evaluation of second derivatives. Furthermore, explicitly quantum effects such as tunneling and zero-point energy are not yet incorporated into the current formulation, although they could in principle be included through a path-integral treatment on the curved configurational manifold.

These considerations point toward several promising directions for future work. One natural extension is the development of Riemannian gradient flow approaches to describe solvent-influenced dynamics within the same geometric setting. Another is the formulation of a geometric Kramers theory with space-dependent metrics, which may provide a more refined description of activated processes in heterogeneous environments. Additional perspectives include the control of bifurcation phenomena through curvature modulation, the study of quantum-classical transition surfaces using Laplace–Beltrami operators, and the extension of the formalism to Finsler-Randers geometries in order to account for velocity-dependent forces such as magnetic interactions or dissipative effects.

In summary, we have proposed an alternative Riemannian formulation of reaction dynamics in which molecular configuration space is treated as a differentiable manifold endowed with a PES-induced metric. This perspective does not modify the underlying physics of chemical reactions; rather, it reframes them in a geometry that makes their intrinsic structure explicit. As in Feynman’s reformulation of quantum mechanics via path integrals, changing the mathematical viewpoint reveals features that remain hidden in traditional formulations.

Within this geometric framework, reaction paths arise as geodesics, while the second variation of the action provides a rigorous coordinate-free stability index directly linked to the Hessian spectrum and the sectional curvature of the PES. This unifies the topography of the energy landscape, variational stability, and pathway selection within a single differential-geometric language. The resulting stability maps offer a detailed diagnostic of hyperbolic (unstable), parabolic (transition), and elliptic (stable) regions, enabling direct identification of transition states, channel rigidity, and the onset of bifurcations. The observed correlation among curvature eigenvalues, vibrational frequencies, and barrier structures further reinforces the physical consistency of the approach.

Beyond recovering known reaction paths and spectroscopic observables with high accuracy, the geometric formulation provides a foundation for several promising extensions, including Riemannian gradient flows for overdamped or solvent-influenced dynamics, geometric Kramers theory with space-dependent metrics, bifurcation-based control of reactivity through curvature modulation, and the characterization of quantum-classical transition surfaces via effective Laplace–Beltrami operators and decoherence metrics. These directions collectively point to a unified geometric framework for predicting, optimizing, and controlling chemical reactivity, transforming reaction path analysis into a problem of tailored geometric design.

Addressing the computational challenge of obtaining fully self-consistent solutions to the field equations will require the development of efficient numerical strategies. We envision three complementary approaches: (i) iterative fixed-point or Newton–Raphson schemes, analogous to those used in numerical relativity, accelerated by using the ordinary Hessian as an initial guess; (ii) finite-element discretization of the configuration space, focusing computational effort on regions of high curvature, such as transition states and bifurcation points; and (iii) machine-learning approaches, where neural networks are trained to approximate the self-consistent metric using the Hessian as input and the Ricci curvature as a loss function. This latter strategy is particularly attractive for large systems as it bypasses the need for explicit iterative solution at every configuration point.

The practical applicability of the RRD framework to larger and more complex reactive systems—particularly catalytic processes involving transition-metal centers—will depend critically on efficient evaluation of the PES and its derivatives. We believe that machine-learned potentials (e.g., neural network potentials, Gaussian approximation potentials, or equivariant message-passing architectures) offer a feasible and scalable strategy. These potentials provide rapid and accurate evaluations of energies and their derivatives, enabling the computation of Hessians (and hence the effective metric) for systems with hundreds or thousands of atoms. Moreover, the RRD framework can actively guide the training process by identifying regions of high curvature that require denser sampling, thereby improving the efficiency and accuracy of the ML potential. The geodesic equation itself can be integrated numerically using automatic differentiation to compute Christoffel symbols, circumventing the need for analytical derivatives of the metric. This synergy between machine learning and differential geometry holds great promise for extending the RRD approach to realistic catalytic and biochemical systems.

## Supplementary Material



## Notes

IRC and RRD—steepest-descent path in mass-weighted Euclidean space versus geodesic in a Hessian-modified Riemannian space (*g*_μν_ = δ_μν_ + α*H*_μν_), external force (∇*V*) versus intrinsic geometry (*R*_μν_), and the two distinct criteria for identifying the transition state; the series expansion of the metric and of the potential energy surface around the equilibrium geometry (*r*_1_,*r*_2_,*r*_3_,θ), arriving at the explicit expression for the second variation of the action δ^2^ *S*; its compact matrix form with the tensors *G* and *K*, and the stability criterion based on the positive-definiteness of *K*, together with the physical interpretation of the diagonal terms (independent harmonic oscillators), the off-diagonal couplings (*k*_12_,*k*_*r*θ_), and the effective angular mass *I*_eff_ = (*m_H_*/2)*R* ^2^; a further section situates this work relative to established approaches, including the Reaction Path Hamiltonian of Miller–Handy–Adams, the Maupertuis–Jacobi principle, and Fukui’s use of IRC curvature, and this is followed by an analysis of the Hessian curvature structure at the two transition states (H_2_O → H + OH and H_2_O → H_2_ + O); Tables S3–S9 compare calculated and experimental values for vibrational frequencies, rotational and anharmonicity constants, structural parameters, and characteristic energies (ZPE, barriers, OH bond energy), alongside normal-mode eigenvectors, and the geometric constants of the Riemannian model (κ, Λ_chem_, *tr*(*g*), det­(*g*), scalar *R*) (PDF)
