# Supplementary material for: A Riemannian Framework for Chemical Reaction Dynamics: Geodesics, Curvature, and Transition-State Stability
Source: ACS Omega. 2026 Oct 16;11(43):66490–8. doi: 10.1021/acsomega.6c05645 (PMC13736977; doi:10.1021/acsomega.6c05645)
Supplement: Supplementary file 1 [file ao6c05645_si_001.pdf]

SUPPORT INFORMATION

for

A Riemannian Framework for Chemical Reaction Dynamics: Geodesics, Curvature, and Transition-State Stability

Kleber C. Mundim [1]*

[1] Universidade Estadual de Goiás, Molecular Sciences Graduate Program, Anápolis, GO, 75132-400, Brazil

* kmundim@gmail.com

Table S1: Geometric correspondence between the Intrinsic Reaction Coordinate (IRC) and the geodesic paths arising from Riemannian Reaction Dynamics (RRD).

Feature	Intrinsic Reaction Coordinate (IRC)	Riemannian Reaction Dynamics (RRD)
**Path Definition**	Steepest descent path (Gradient flow) in mass-weighted Euclidean space.	Geodesic (Path of extremal action) in a Hessian-modified Riemannian space.
**Metric ($g_{\mu\nu}$)**	$g_{\mu\nu} \propto \delta_{\mu\nu}$ (Mass-weighted Euclidean).	$g_{\mu\nu} = \delta_{\mu\nu} + \alpha H_{\mu\nu}$ (Hessian-modified).
**Dynamics Source**	External Force (Gradient ∇V)	Intrinsic Geometry (Curvature $R_{\mu\nu}$)
**Transition State**	First-order saddle point (defined by $\nabla V = 0$ and $\widetilde{H}$ eigenvalues).	First-order saddle point (defined by $\delta^2 S < 0$ and $\mu_n < 0$ in the Jacobi equation).

1. **Derivation Geodesic Equation for a potential $V(q)$:**

a. Series Expansion Around the Equilibrium Point

We consider small deviations around the equilibrium geometry $\mathbf{q}_0 = (r_1^0, r_2^0, r_3^0, \theta^0)$ of the water molecule:

$$\mathbf{q} = \mathbf{q}_0 + \delta\mathbf{q}, \quad \delta\mathbf{q} = (\delta r_1, \delta r_2, \delta r_3, \delta\theta)$$

At the equilibrium point, the metric tensor becomes constant:

$$g_{ij}(\mathbf{q}_0) = \begin{pmatrix} m_H & 0 & 0 & 0 \\ 0 & m_H & 0 & 0 \\ 0 & 0 & \frac{m_H}{2} & 0 \\ 0 & 0 & 0 & \frac{m_H}{2}[(r_1^0)^2 + (r_2^0)^2] \end{pmatrix}$$

b. Expansion of the Potential Energy

The Potential Energy Surface (PES) $V(\mathbf{q})$ is expanded to second order:

$$V(\mathbf{q}_0 + \delta\mathbf{q}) = V(\mathbf{q}_0) + \sum_i \frac{\partial V}{\partial q^i}|_{\mathbf{q}_0} \delta q^i + \frac{1}{2}\sum_{i,j} \frac{\partial^2 V}{\partial q^i \partial q^j}|_{\mathbf{q}_0} \delta q^i \delta q^j + \mathcal{O}(\delta\mathbf{q}^3)$$

At equilibrium, the first derivatives vanish:

$$\frac{\partial V}{\partial q^i}|_{\mathbf{q}_0} = 0 \quad \text{for } i = 1,2,3,4$$

Thus, the expansion simplifies to:

$$V(\mathbf{q}_0 + \delta\mathbf{q}) = V(\mathbf{q}_0) + \frac{1}{2}\sum_{i,j} V_{ij}\,\delta q^i \delta q^j + \mathcal{O}(\delta\mathbf{q}^3)$$

where $V_{ij} = \frac{\partial^2 V}{\partial q^i \partial q^j}|_{\mathbf{q}_0}$ is the Hessian matrix.

c. Expanded Lagrangian and Second Variation of the Action

The Lagrangian $L = T - V$ becomes:

$$L = \frac{1}{2}\sum_{i,j} g_{ij}\,\delta\dot{q}^i \delta\dot{q}^j - V(\mathbf{q}_0) - \frac{1}{2}\sum_{i,j} V_{ij}\,\delta q^i \delta q^j + \mathcal{O}(\delta\mathbf{q}^3)$$

The second variation of the action, $\delta^2 S$, which is quadratic in the fluctuations, is then:

$$\delta^2 S = \frac{1}{2}\int_{t_1}^{t_2}\left[\sum_{i,j} g_{ij}\,\delta\dot{q}^i \delta\dot{q}^j - \sum_{i,j} V_{ij}\,\delta q^i \delta q^j\right] dt$$

d. Explicit Calculation of the Terms

Kinetic Energy Term

$$T_{\text{kin}} = \frac{1}{2}\left[m_H(\delta\dot{r}_1)^2 + m_H(\delta\dot{r}_2)^2 + \frac{m_H}{2}(\delta\dot{r}_3)^2 + \frac{m_H}{2}[(r_1^0)^2 + (r_2^0)^2]\left(\delta\dot{\theta}\right)^2\right]$$

Potential Energy Term and the Hessian Matrix

For the given PES:

$$\begin{aligned} V = \quad & D_{e,\text{OH}}\sum_{i=1}^{2}\left(1 - e^{-a_{\text{OH}}(r_i - r_{e,\text{OH}})}\right)^2 + D_{e,\text{HH}}\left(1 - e^{-a_{\text{HH}}(r_3 - r_{e,\text{HH}})}\right)^2 \\ & + \frac{1}{2}k_\theta(\theta - \theta_e)^2 + k_c\left[\left(r_1 - r_{e,\text{OH}}\right) + \left(r_2 - r_{e,\text{OH}}\right)\right](\theta - \theta_e) - Ae^{-\Phi/\sigma^2} - D_{e,\text{OH}} \end{aligned}$$

At equilibrium $\left(r_1^0 = r_2^0 = r_{e,\text{OH}}, \theta^0 = \theta_e\right)$, the relevant second derivatives are:

- **Diagonal Force Constants:**

$$\frac{\partial^2 V}{\partial r_1^2} = \frac{\partial^2 V}{\partial r_2^2} = 2D_{e,\text{OH}} a_{\text{OH}}^2 + \frac{\partial^2 V_{\text{exp}}}{\partial r_1^2} \equiv k_r$$

$$\frac{\partial^2 V}{\partial r_3^2} = 2D_{e,\text{HH}} a_{\text{HH}}^2 + \frac{\partial^2 V_{\text{exp}}}{\partial r_3^2} \equiv k_{r_3}$$

$$\frac{\partial^2 V}{\partial \theta^2} = k_\theta + \frac{\partial^2 V_{\text{exp}}}{\partial \theta^2} \equiv k_\theta$$

- **Off-Diagonal Coupling Constants:**

$$\frac{\partial^2 V}{\partial r_1\,\partial r_2} = \frac{\partial^2 V_{\text{exp}}}{\partial r_1\,\partial r_2} \equiv k_{12}$$

$$\frac{\partial^2 V}{\partial r_1\,\partial \theta} = \frac{\partial^2 V}{\partial r_2\,\partial \theta} = k_c + \frac{\partial^2 V_{\text{exp}}}{\partial r_1\,\partial \theta} \equiv k_{r\theta}$$

The derivatives of the exponential term $V_{\text{exp}} = -Ae^{-\Phi/\sigma^2}$ are typically small at equilibrium if Φ is not near zero.

e. Final Expression for $\delta^2 S$

Substituting the metric and the Hessian, the final expression is:

$$\delta^2 S = \frac{1}{2}\int_{t_1}^{t_2} [\quad m_H(\delta\dot{r}_1)^2 + m_H(\delta\dot{r}_2)^2 + \frac{m_H}{2}(\delta\dot{r}_3)^2 + \frac{m_H}{2}R^2\left(\delta\dot{\theta}\right)^2$$
$$-k_r(\delta r_1)^2 - k_r(\delta r_2)^2 - k_{r_3}(\delta r_3)^2 - k_\theta(\delta\theta)^2$$
$$-2k_{12}\delta r_1\delta r_2 - 2k_{r\theta}\delta r_1\delta\theta - 2k_{r\theta}\delta r_2\delta\theta]dt$$

where $R^2 = (r_1^0)^2 + (r_2^0)^2$.

f. Compact Matrix Form

The expression can be written in a compact matrix form:

$$\delta^2 S = \frac{1}{2}\int_{t_1}^{t_2} [\delta\dot{\mathbf{q}}^T \cdot \mathbf{G} \cdot \delta\dot{\mathbf{q}} - \delta\mathbf{q}^T \cdot \mathbf{K} \cdot \delta\mathbf{q}]\,dt$$

With the constant mass-metric tensor $\mathbf{G}$ and force-constant matrix $\mathbf{K}$:

$$\mathbf{G} = \begin{pmatrix} m_H & 0 & 0 & 0 \\ 0 & m_H & 0 & 0 \\ 0 & 0 & \frac{m_H}{2} & 0 \\ 0 & 0 & 0 & \frac{m_H}{2}R^2 \end{pmatrix}, \quad \mathbf{K} = \begin{pmatrix} k_r & k_{12} & 0 & k_{r\theta} \\ k_{12} & k_r & 0 & k_{r\theta} \\ 0 & 0 & k_{r_3} & 0 \\ k_{r\theta} & k_{r\theta} & 0 & k_\theta \end{pmatrix}$$

g. Physical Interpretation and Applications

This result describes the system's dynamics for small fluctuations around equilibrium:

-Diagonal Terms: Represent independent harmonic oscillators for each coordinate.

- Off-Diagonal Couplings **(**$k_{12}, k_{r\theta}$**)**: Describe how fluctuations in different coordinates influence each other (e.g., bond stretch-angle coupling).

- Effective Angular Mass: The term $I_{\text{eff}} = \frac{m_H}{2} R^2$ acts as the moment of inertia for bending motions.

- Stability Criterion: The equilibrium is stable if the matrix $\mathbf{K}$ is positive definite (all eigenvalues > 0).

This harmonic expansion is the foundation for calculating:

- Vibrational Frequencies: By diagonalizing $\mathbf{G}^{-1}\mathbf{K}$.
- Thermal Fluctuations: Using the equipartition theorem.
- Quantum Fluctuations & Correlation Functions: Via functional integration over the paths $\delta\mathbf{q}(t)$.
- Franck-Condon Factors: In spectroscopic transitions.

This approximation is valid for small amplitude motions where higher-order (anharmonic) terms in the PES expansion are negligible.

Comparison with existing approaches

The use of Riemannian geometry to study chemical dynamics is not new; it has a significant and well-established history in theoretical chemistry:

- **Reaction Path Hamiltonian (RPH) / IRC:** The RPH, introduced by Miller, Handy, and Adams, uses a Riemannian metric (specifically, the mass-weighted Euclidean metric) to define the reaction path (IRC) and to decouple motion along the path from motion perpendicular to it. The RPH formalism itself is a manifestation of the underlying Riemannian geometry of configuration space.
- **Jacobi Metric Dynamics:** As discussed earlier, the Maupertuis–Jacobi principle naturally introduces a metric in which trajectories are geodesics. The RRD concept of reaction paths as geodesics is a direct geometric extension of this classical principle, although RRD employs a unique and more complex metric linked to the Hessian. In the Jacobi framework, this is expressed as ($g_J = 2(E - V)\, g_M$).
- **IRC Inflection Point:** Early work by Fukui and others used the curvature of the IRC to analyze reaction features.

Feature	Intrinsic Reaction Coordinate (IRC)	Riemannian Reaction Dynamics (RRD)
**Path**	Steepest-descent path (gradient	Geodesic (extremal-action path) in a

**definition**	flow) in mass-weighted Euclidean space.	Hessian-modified Riemannian space.
**Metric $g_{\mu\nu}$**	$g_{\mu\nu} \propto \delta_{\mu\nu}$ mass-weighted Euclidean).	$g_{\mu\nu} = \delta_{\mu\nu} + \alpha H_{\mu\nu}$ (Hessian-modified).
**Source of dynamics**	External force (gradient) (∇V)	Intrinsic geometry (curvature) ($R_{\mu\nu}$)
**Transition state**	First-order saddle point (defined by $\nabla V = 0$ and the eigenvalues of $\widetilde{H}$.	First-order saddle point (defined by $\delta^2 S < 0$ and $\mu_n < 0$ in the Jacobi equation).

SI Table 2: Comparison with existing approaches.

Physical Interpretation

For the transition state $H_2O \rightarrow H + OH$:

- **Diagonal elements** (units: kJ/mol·Å² for bond distances; kJ/mol·rad² for angular distortions):
  $H_{r_1r_1}$: curvature along the OH_1 bond
  $H_{r_2r_2}$: curvature along the OH_2 bond
  $H_{r_3r_3}$: curvature along the HH coordinate
  $H_{\theta\theta}$: angular curvature (smaller than bond-distortion terms
- **Off-diagonal elements:**
  $H_{r_1r_2}$: coupling between the two OH bonds
  $H_{r_1r_3}$: coupling between OH_1 and HH
  $H_{r_2r_3}$: coupling between OH_2 and HH
  $H_{r_1\theta}$: coupling between OH_1 and the bending angle
  $H_{r_2\theta}$: coupling between OH_2 and the bending angle
  $H_{r_3\theta}$: no direct coupling between HH and the angle

For the transition state $H_2O \rightarrow H_2 + O$:

1. **Reaction direction:**
   Very small curvature along r2r_2r2 (≈46) indicates that this coordinate defines the primary reaction direction, corresponding to the elongation of the second OH bond.

2. **Narrow/unstable mode:**
   A small negative curvature leads to an imaginary frequency along r2r_2r2, while all other coordinates display positive curvature (stable modes).
3. **Coupling structure:**
   Off-diagonal terms are reduced compared to the first transition state.
   The system becomes more "decoupled" due to bond breaking.
4. **Intrinsic geometry:**
   The configuration space becomes highly anisotropic.
   The "geometric distance" between configurations varies strongly with direction.

Table with constants

SI Table S3. Fundamental Vibrational Frequencies

Vibrational Mode	Calculated (cm^{-1})	Experimental (cm^{-1})	Deviation	Symmetry
Symmetric OH Stretch	3825	3657	+4.6%	A_1
Asymmetric OH Stretch	3715	3756	−1.1%	B_2
HOH Bending	1595	1595	0.0%	A_1

Reference: Herzberg, Molecular Spectra and Molecular Structure.

SI Table S4. Rotational Constants

Constant	Calculated	Experimental	Deviation	Units
B_e (OH)	18.9	18.9	0.0%	cm^{-1}
A_e (H_2O)	27.8	27.9	−0.4%	cm^{-1}
C_e (H_2O)	9.3	9.4	−1.1%	cm^{-1}
Inertia Moment (I_a)	1.02×10^{-47}	1.01×10^{-47}	+1.0%	$kg{\cdot}m^2$

SI Table S5. Anharmonicity Constants

Constant	Calculated	Experimental	Deviation
$\omega_e \chi_e$ (OH)	82.5	80.4	+2.6%
α_e (OH)	0.724	0.707	+2.4%
γ_e (OH)	0.015	0.016	−6.3%
χ_{11} (HOH)	−42.3	−43.9	−3.6%

SI Table S6. Equilibrium Structural Parameters

Parameter	Calculated	Experimental	Deviation	Units
r_e (OH)	0.9572	0.9572	0.0%	Å
θ_e (HOH)	104.5	104.5	0.0%	degree
r_e (HH)	1.514	1.515	–0.1%	Å
OH Dissociation Energy	497.1	497.1	0.0%	kJ/mol

SI Table S7. Riemannian Model Constants

Geometric Parameter	Value	Physical Interpretation	Units
κ (coupling constant)	0.00345	Curvature–frequency scaling	dimensionless
Λ(_{chem})	0.0237	"Chemical cosmological constant"	dimensionless
tr(g)	2646.2	"Volume" of configuration space	kJ/mol
det(g)	3.21×10^{8}	Determinant of the metric	$(kJ/mol)^4$
Scalar Curvature R	0.045	Mean curvature at equilibrium	$Å^{-2}$
Geometric Parameter	Value	Physical Interpretation	Units

SI Table S8. Characteristic Energies

Energy (kJ/mol)	Calculated	Experimental
Zero-Point Energy (ZPE)	55.3	56.4
Barrier H_2O → H + OH	499.2	~500
Barrier H_2O → H_2 + O	492.8	~495
OH Bond Energy	459.8	458.9

SI Table S9. Normal Modes — Metric Eigenvectors

Coordinate	Mode 1 (3825 cm^{-1})	Mode 2 (3715 cm^{-1})
r_1 (OH_1)	0.707	0.707
r_2 (OH_2)	0.707	0.707
r_3 (HH)	0.000	0.000
θ (HOH)	0.000	0.000
